# Influenza Virus Reassortment Occurs with High Frequency in the Absence of Segment Mismatch

**DOI:** 10.1371/journal.ppat.1003421

**Published:** 2013-06-13

**Authors:** Nicolle Marshall, Lalita Priyamvada, Zachary Ende, John Steel, Anice C. Lowen

**Affiliations:** 1 Department of Microbiology and Immunology, Emory University School of Medicine, Atlanta, Georgia, United States of America; 2 Microbiology and Molecular Genetics Graduate Program, Emory University, Atlanta, Georgia, United States of America; 3 Emory Vaccine Center at Yerkes National Primate Research Center, Atlanta, Georgia, United States of America; 4 Immunology and Molecular Pathogenesis Graduate Program, Emory University, Atlanta, Georgia, United States of America; Mount Sinai School of Medicine, United States of America

## Abstract

Reassortment is fundamental to the evolution of influenza viruses and plays a key role in the generation of epidemiologically significant strains. Previous studies indicate that reassortment is restricted by segment mismatch, arising from functional incompatibilities among components of two viruses. Additional factors that dictate the efficiency of reassortment remain poorly characterized. Thus, it is unclear what conditions are favorable for reassortment and therefore under what circumstances novel influenza A viruses might arise in nature. Herein, we describe a system for studying reassortment in the absence of segment mismatch and exploit this system to determine the baseline efficiency of reassortment and the effects of infection dose and timing. Silent mutations were introduced into A/Panama/2007/99 virus such that high-resolution melt analysis could be used to differentiate all eight segments of the wild-type and the silently mutated variant virus. The use of phenotypically identical parent viruses ensured that all progeny were equally fit, allowing reassortment to be measured without selection bias. Using this system, we found that reassortment occurred efficiently (88.4%) following high multiplicity infection, suggesting the process is not appreciably limited by intracellular compartmentalization. That co-infection is the major determinant of reassortment efficiency in the absence of segment mismatch was confirmed with the observation that the proportion of viruses with reassortant genotypes increased exponentially with the proportion of cells co-infected. The number of reassortants shed from co-infected guinea pigs was likewise dependent on dose. With 10^6^ PFU inocula, 46%–86% of viruses isolated from guinea pigs were reassortants. The introduction of a delay between infections also had a strong impact on reassortment and allowed definition of time windows during which super-infection led to reassortment in culture and in vivo. Overall, our results indicate that reassortment between two like influenza viruses is efficient but also strongly dependent on dose and timing of the infections.

## Author Summary

Reassortment is the process by which influenza viruses, which carry RNA genomes comprising eight segments, exchange genetic material. Reassortment of the genome segments of two differing influenza strains has the potential to vastly increase the diversity of circulating influenza viruses. Despite its importance to influenza virus evolution, the frequency with which reassortment occurs in a cell or an animal infected with two or more variant viruses is unclear. Toward determining how readily reassortment can occur, we assessed the incidence of reassortment during experimental infection in cultured cells and in guinea pigs. We found that reassortment can occur with high efficiency in both systems, but that that efficiency is dependent on i) the dose of each virus added to the cells or taken up by the host and ii) the relative timing with which each virus infects. These results suggest that influenza A virus reassortment may be more prevalent in nature than one might expect based on the results of surveillance studies.

## Introduction

Reassortment is the process by which viruses carrying segmented genomes exchange gene segments. The reshuffling of genetic material achieved through reassortment supports rapid production of variant viruses that can be markedly different, genotypically and phenotypically, from the parental strains. The more gradual process of genetic drift, resulting from errors in genome replication, and the process of reassortment come together to generate vast genomic diversity among influenza A viruses. It is this diversity that, in turn, permits the rapid evolution of influenza viruses and the generation of novel pandemic and epidemic strains.

The contribution of reassortment to the emergence of pandemic influenza viruses is well established: the 1957 and 1968 pandemics arose following reassortment events between avian and human influenza viruses that allowed novel HA subtypes to gain widespread circulation in the human population [1], [2], [3]. Reassortment furthermore played a prominent role in the creation of the H5N1 viruses that continue to circulate in poultry of Southeast Asia [4], and in the H1N1 swine influenza viruses that emerged in humans in April 2009 [5], [6]. Thus, epidemiological studies indicate that reassortment is an important means of viral diversification and often facilitates inter-species transmission.

In addition to its role in pandemic influenza, phylogenetic studies have revealed the importance of reassortment between co-circulating viruses of the same subtype in generating a diverse pool of seasonal influenza viruses [7], [8], [9], [10], [11], [12], [13], [14]. This diversity in turn allows for the selection of variants that escape pre-existing immunity in the population and thereby cause widespread epidemics: evidence suggests that the unusually severe epidemics of 2003, 1951 and 1947 were each caused by strains generated through intra-subtype reassortment among co-circulating clades [8], [10].

Previous efforts to study influenza virus reassortment in the lab have been of three main types. First, beginning with the work of Lubeck et al. in 1979, several research groups have examined the phenomenon of segment mismatch, in which the gene segments of two differing strains are found to assort in a non-random fashion due to functional incompatibilities between the viral proteins or RNA segments [15], [16], [17], [18]. It is clear from this literature that strain differences between parental viruses limit the fitness of many reassortant progeny and thereby restrict the number of different genotypes that arise, or are detected, following co-infection. Thus, segment mismatch is a potent determinant of reassortment efficiency. Second, a number of risk assessment type studies have addressed the potential for variants with increased virulence or transmissibility to arise through reassortment between two strains of epidemiologic importance [19], [20], [21], [22], [23], [24], [25], [26], [27]. Third, since reassortment between circulating strains and the egg-adapted A/Puerto Rico/8/34 virus has been used since 1969 to generate vaccine seed strains that grow well in embryonated chicken's eggs, significant research effort has been put into optimizing this procedure [28], [29].

Research to date is lacking on the conditions of co-infection that are most favorable for reassortment, and it is therefore unclear under what circumstances we can expect to see novel influenza A viruses arising in nature. In part, this knowledge gap has arisen because, when one studies reassortment between two dissimilar strains, the effects of other parameters are confounded by those of segment mismatch. Herein, we report a novel method for the study of reassortment in the absence of segment mismatch and the application of this method to determine the baseline frequency of reassortment under unbiased conditions, and the impacts of infection dose and timing on this baseline.

## Results

### A system for the study of unbiased reassortment

**Figure 1:**
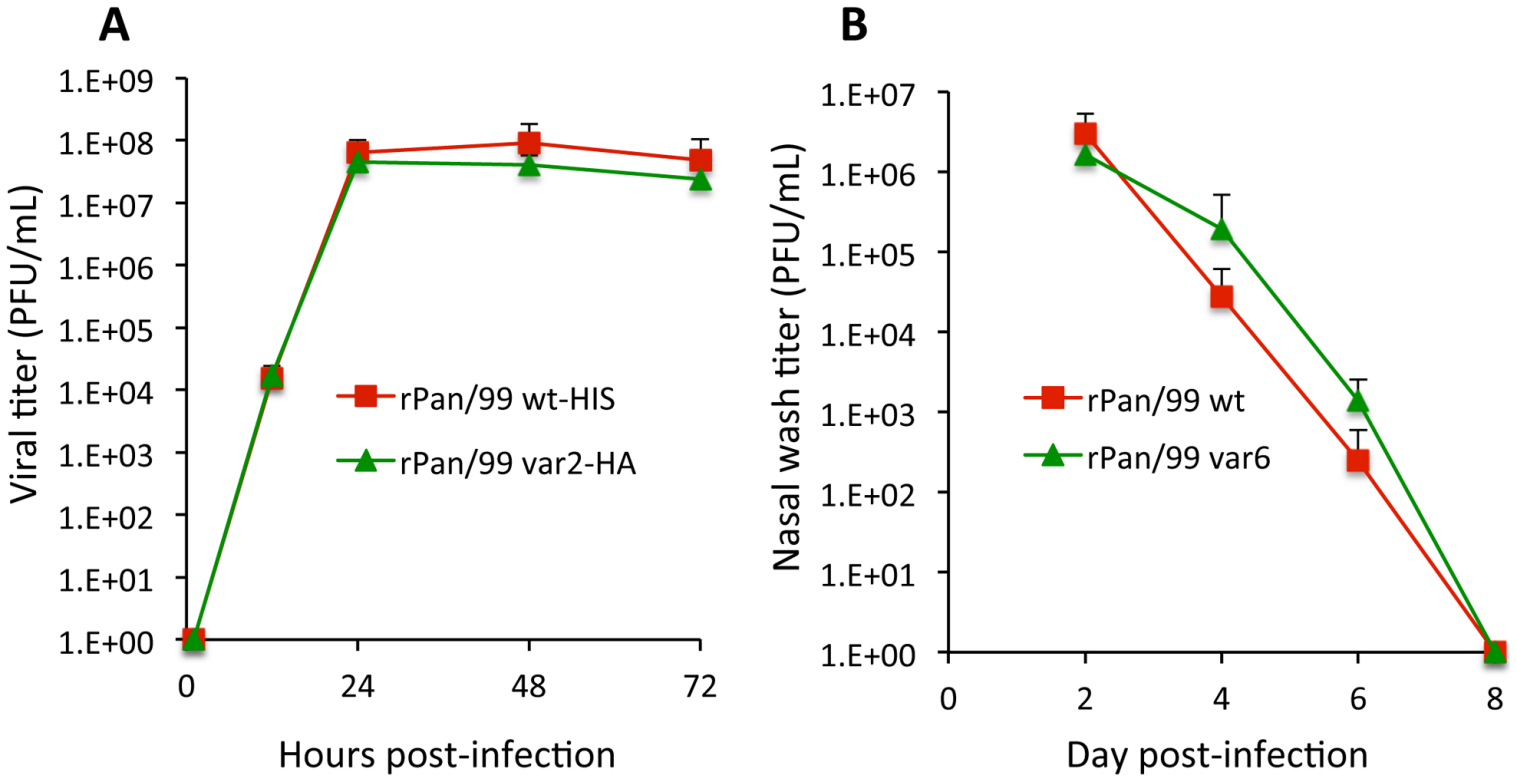
rPan/99 wt and var viruses show similar growth phenotypes in MDCK cells and guinea pigs. A) MDCK cells were infected at an MOI of 0.001 PFU/cell with the indicated viruses. For rPan/99wt-HIS virus, n = 6 dishes; for rPan/99var2-HA virus, n = 3 dishes. B) Groups of three guinea pigs were inoculated intranasally with 1000 PFU of the indicated virus. Virus titers in nasal washings are plotted vs. day post-infection. Average values +/− standard deviations are shown.

In order to obtain data that are not confounded by segment mismatch, we have designed an approach that employs a pair of phenotypically identical but genotypically distinct influenza viruses. Reverse genetics was used to introduce silent mutations into each gene segment of A/Panama/2007/99 (H3N2) [Pan/99] virus such that the segments of the resultant variant, or rPan/99var, virus can be distinguished from those of the rPan/99wt virus using molecular techniques (described below). The mutations introduced were selected carefully such that the rPan/99var viruses were not attenuated in growth relative to the rPan/99wt strain (Figure 1). As a result, all 256 different progeny that might arise following co-infection with rPan/99wt and rPan/99var viruses are expected to be of equal fitness. Because there are no selective pressures acting differentially on the various progeny strains, co-infection with rPan/99wt and rPan/99var viruses constitutes an unbiased system in which to study reassortment.

**Figure 2:**
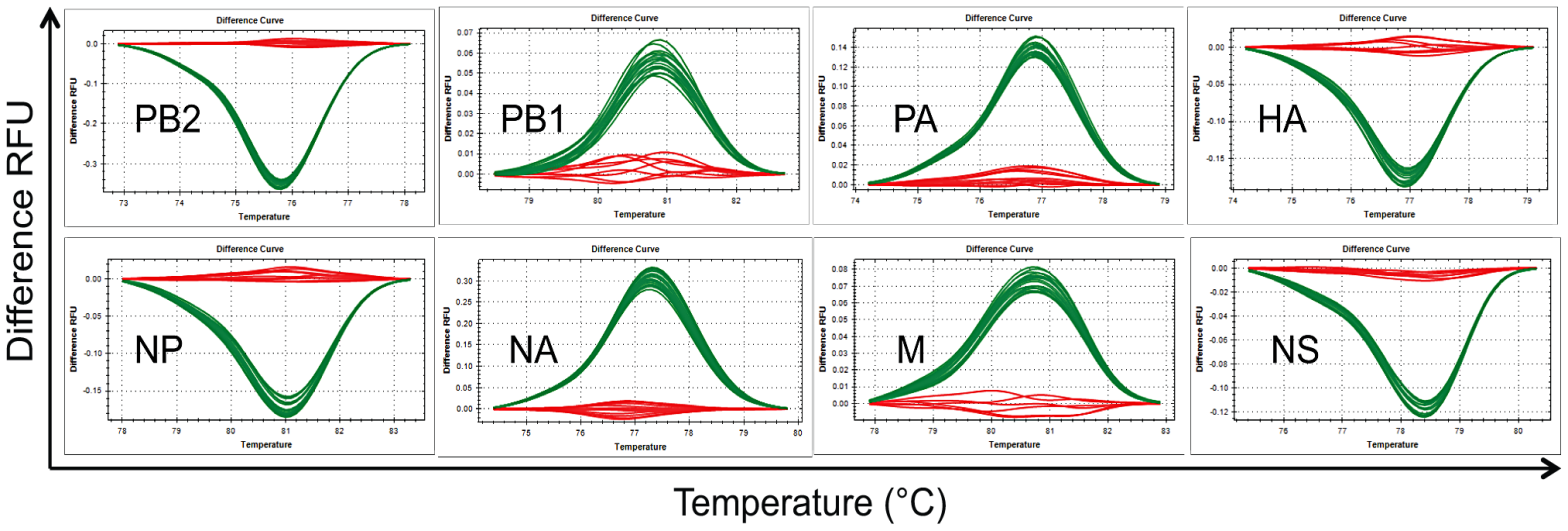
Identification of wild-type and variant virus gene segments by high resolution melt analysis. Examples of the Difference RFU (relative fluorescence units) curves generated by the Precision Melt Analysis software are shown for each vRNA segment. Curves colored red clustered with the rPan/99wt control and curves colored green clustered with the rPan/99var control.

As shown in Figure 2, the silent mutations differentiating rPan/99wt and rPan/99var viruses allow the full genotypes of progeny arising from mixed infections to be determined using high resolution melt (HRM) analysis [30]. This method exploits the fact that sequence differences between two double stranded DNA (dsDNA) molecules confer differences in melting properties. These differences in melting properties can in turn be detected as changes in fluorescence when dsDNAs labelled with a saturating fluorescent dye are heated (e.g. from 65°C to 95°C), since the dye will cease to fluoresce as the DNA melts into single strands. As described in more detail in the methods section, we have applied HRM analysis to clonal virus isolates derived from co-infection in order to identify each gene segment as either wt or var in origin.

### Reassortment occurs with high efficiency under unbiased conditions

**Figure 3:**
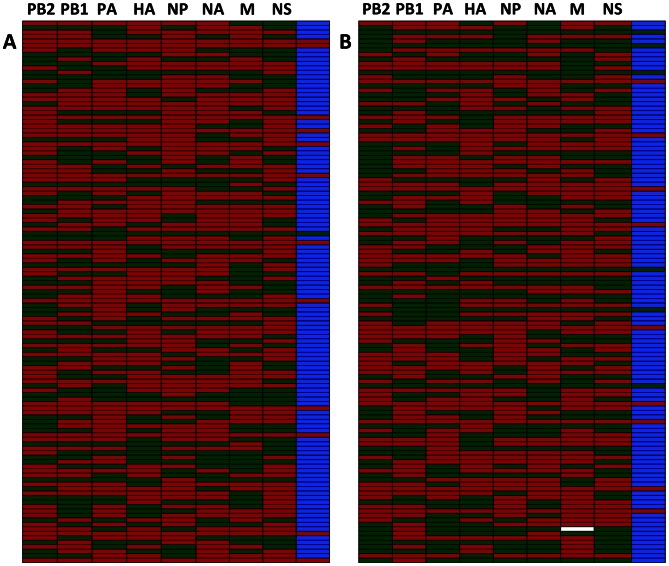
Reassortment in cultured cells is efficient under unbiased conditions. The results are shown of HRM genotyping analysis of 121 plaque isolates obtained from each of two culture dishes of MDCK cells co-infected at high MOI (10 PFU/cell). In A, 108/121 (89.3%) had reassortant genotypes and in B, 106/121 (87.6%) had reassortant genotypes. This experiment was done in the presence of trypsin. Green coloring indicates a segment derived from rPan/99var virus; red coloring indicates a segment derived from Pan/99wt virus; white indicates a segment that was untyped. The right-most column in each chart shows the overall genotype: green for var, red for wt and blue for reassortant.

We have defined the baseline frequency of reassortment as the percentage of progeny viruses with reassortant genotypes that arises after a single cycle of replication, given high levels of co-infection at the cellular level and an absence of segment mismatch or other selection pressures that would promote parental genotypes over reassortant ones. To determine this baseline value, rPan/99wt-HIS and rPan/99var-HA viruses were used to co-infect MDCK cells at a multiplicity of infection of 10 PFU/cell of each virus. Epitope tagged versions of the wt and var strains were used so that the number of cells infected with each virus and the number of cells co-infected could be determined by flow cytometry. Co-infection rates of 99.4% were achieved in each of two independent samples. As shown in Figure 3, the resultant frequencies of reassortment were 87.6% and 89.2% (average = 88.4%). While this result indicates that reassortant viruses arise with high frequency under the unbiased conditions described, the theoretically optimal efficiency of 254/256, or 99.2%, was not achieved: parental progeny viruses were over-represented relative to the expected frequency of 0.8% (p<0.001, exact test). This discrepancy could be suggestive of incomplete mixing between genomes in co-infected cells, but also may be the result of a small differences in the input of wt and var viruses into individual cells, due either to a slight skewing of the inoculum or to the Poisson distribution (with MOIs of 10, a sizable proportion of cells would be expected to receive, for example, 10 copies of the wt virus but 9 or 11 copies of the var virus).

### The frequency of reassortant progeny shows an exponential relationship with the frequency of co-infected cells

**Figure 4:**
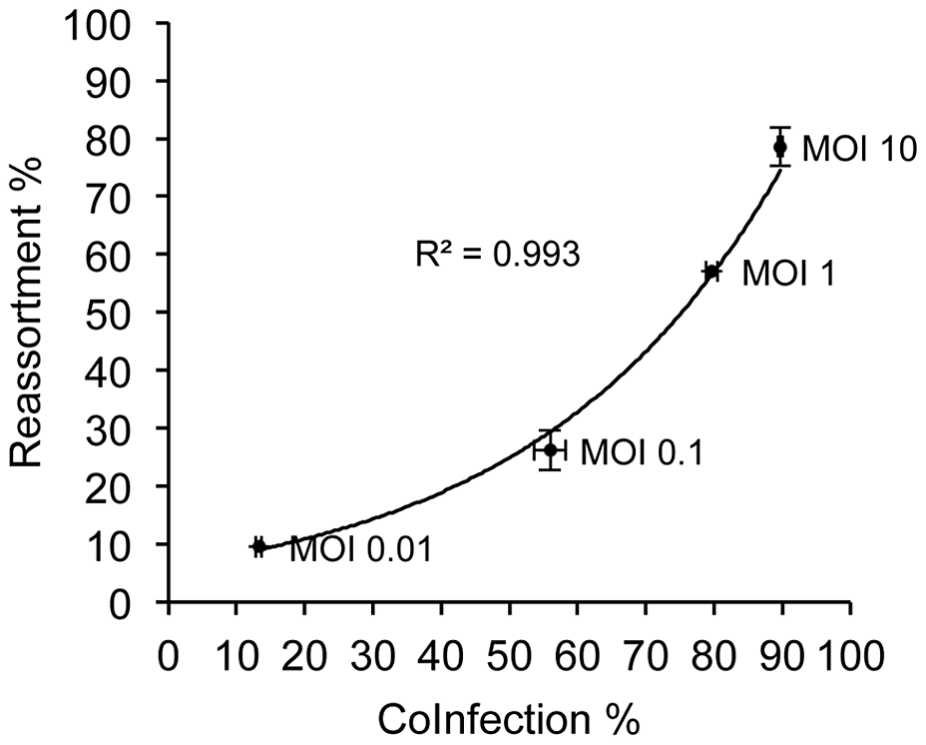
Frequency of reassortment increases exponentially with frequency of co-infection. MDCK cells were infected simultaneously at the indicated MOIs with rPAN/99wt and rPan/99var viruses carrying His and HA tags, respectively. At 12 h post-infection, cells were collected for flow cytometric analysis to determine co-infection frequency and cell culture medium was collected for genotyping of released virus. To prevent multiple cycles of replication, infections were performed in the absence of trypsin. Average values (n = 2 cell culture dishes) +/− standard deviation are shown. The coefficient of determination (*R* ^2^) value indicates how well the exponential trend line fits the data.

Co-infection at the cellular level is a necessary precursor to reassortment. To determine the quantitative relationship between the frequency of co-infected cells and frequency of reassortant progeny viruses, we examined both co-infection and reassortment levels following inoculation of MDCK cells at a range of multiplicities from 10 PFU/cell to 0.01 PFU/cell. Twelve hours after infection with rPan/99wt and rPan/99var viruses, supernatant was collected to determine the frequency of reassortant viruses therein. At the same time, cells were harvested and used to determine the numbers of uninfected, singly infected and doubly infected cells by flow cytometry. As shown in Figure 4, co-infection of MDCK cells was seen with all four MOI conditions but spanned a wide range, with about 13% of infected cells harbouring both wt and var viruses at the lowest MOI to about 90% at the highest MOI. Reassortment was also seen following infection at all four multiplicities and, as predicted, occurred with the highest frequency (average 78.5%) at the highest MOI and the lowest frequency (average 9.5%) at the lowest MOI. In particular, reductions in the proportion of reassortant progeny were significant when the MOI was reduced from 10 to 0.1 or 0.01 PFU/cell (p<0.0001, Fisher's exact test). Rather than a simple linear relationship, however, the data suggest that reassortment levels increased exponentially with the proportion of cells that were co-infected (Figure 4).

### The frequency of reassortant progeny arising in vivo is dependent on inoculum dose

The results obtained following co-infection of cultured cells at a range of MOIs indicated that, as expected, reassortment levels are acutely dependent on co-infection rates. In an animal host, one might expect co-infection and therefore reassortment rates to be low since the number of epithelial cells available for viral infection within the respiratory tract is presumably high. To address this hypothesis, guinea pigs were co-infected intranasally with rPan/99wt and rPan/99var viruses at two doses: 10^3^ PFU or 10^6^ PFU of each strain. At 48 h post-infection nasal lavage was collected from each guinea pig and viruses therein were genotyped. As reported in Table 1, robust reassortment levels were detected under both conditions (averages of 30% and 59% following infection with 10^3^ PFU and 10^6^ PFU, respectively). The difference between the two dosage groups was found to be significant (p = 0.03, Student's t-test), indicating that the level of reassortment in vivo is dependent on inoculum dose.

**Table 1:** The frequency of reassortment in vivo is dependent on inoculum dose^1^.

Guinea pig no.	Inoculum dose (log_10_PFU/ml)	Input mixture^2^		Genotypes of virus isolates (%)^3^		
		% wt	% var	Reassortant	wt	Var
1	3	60	40	24	67	9
2	3	60	40	53	47	0
3	3	60	40	10	86	5
4	3	52	48	20	70	10
5	3	52	48	42	32	26
6	6	50	50	65	5	30
7	6	50	50	46	0	54
8	6	50	50	86	0	14
9	6	54	46	48	48	5
10	6	54	46	48	48	5

1 The difference in % reassortants between 10^3^ and 10^6^ groups was found to be significant, with p = 0.03 (Student's T-test).

2 n was 30 for 1, 2, 3, 6, 7, and 8; n was 25 for 4 and 5; n was 26 for 9 and 10.

3 n ranged from 19 to 24.

### Super-infection up to 8 hours after primary infection led to robust reassortment in cell culture

**Figure 5:**
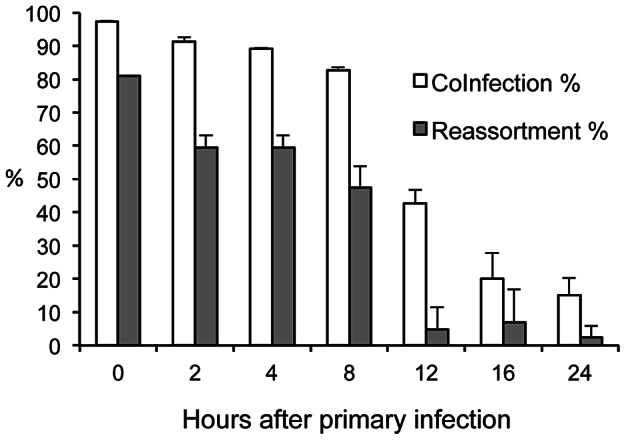
Super-infection delayed by up to 12 h allowed robust co-infection and by up to 8 h allowed robust reassortment in cell culture. MDCK cells were infected at MOI 10 PFU/cell with rPan/99var2-HA virus at 0 h and then super-infected at MOI 10 PFU/cell with rPan/99wt-HIS virus after the indicated time interval. Clonal isolates from supernatant collected 12 h after the wt virus infection were obtained and genotyped to determine the % reassortment. Flow cytometry was performed on the harvested cells to determine % co-infection. To prevent multiple cycles of replication, infections were performed in the absence of trypsin. Average values (n = 2 cell culture dishes) +/− standard deviations are shown.

In nature, the probability of an individual being co-infected with two distinct strains (as opposed to a quasispecies) of influenza virus simultaneously is expected to be quite low; rather, most co-infections are likely to arise from sequential infection events. We therefore sought to determine the window of time during which a second infection can result in reassortment. To this end, experiments were performed in which inoculation of MDCK cells with rPan/99var virus was followed at a range of time points by inoculation with rPan/99wt virus. MDCK cells were infected with either rPan/99var2-HA virus and rPan/99wt-HIS virus together (0 h) or with rPan/99var2-HA virus alone, followed by sequential infections with rPan/99wt-HIS virus 2, 4, 8, 12, 16 or 24 h later. Following each addition of virus, a 1 h attachment period at 4°C was used to synchronize infections; since the viral neuraminidase would not be active at this temperature, it is important to note that any stripping of sialic acids from infected cell surfaces would occur up to the point when the second virus was added, but not during the attachment period. At 12 h post-infection with rPan/99wt-HIS virus, supernatant was collected for genotyping of released virus and cells were harvested to determine co-infection rates by flow cytometry. The results show (Figure 5) that a delay of up to 8 h between the primary and secondary infection led to a 5–16% decrease in the proportion of infected cells that were co-infected when compared to a simultaneous infection. These moderate decreases in the number of co-infected cells were accompanied by larger decreases in the amount of reassortment (41% reduction in the 8 h group relative to the 0 h group, p = 0.003, Fisher's exact test). Nevertheless, up to an 8 h time interval between additions of rPan/99wt and rPan/99var viruses allowed for robust reassortment: 47.5% of virus isolates sampled from the 8 h infections carried a mixed genotype. In contrast, a delay of 12 h between primary and secondary infections resulted in just 4.75% of isolates showing reassortment. This low level of reassortment with the 12 h interval occurred despite the fact that 43% of infected cells were doubly infected under these conditions.

### Super-infection up to 12 hours after primary infection led to robust reassortment in the guinea pig model

**Figure 6:**
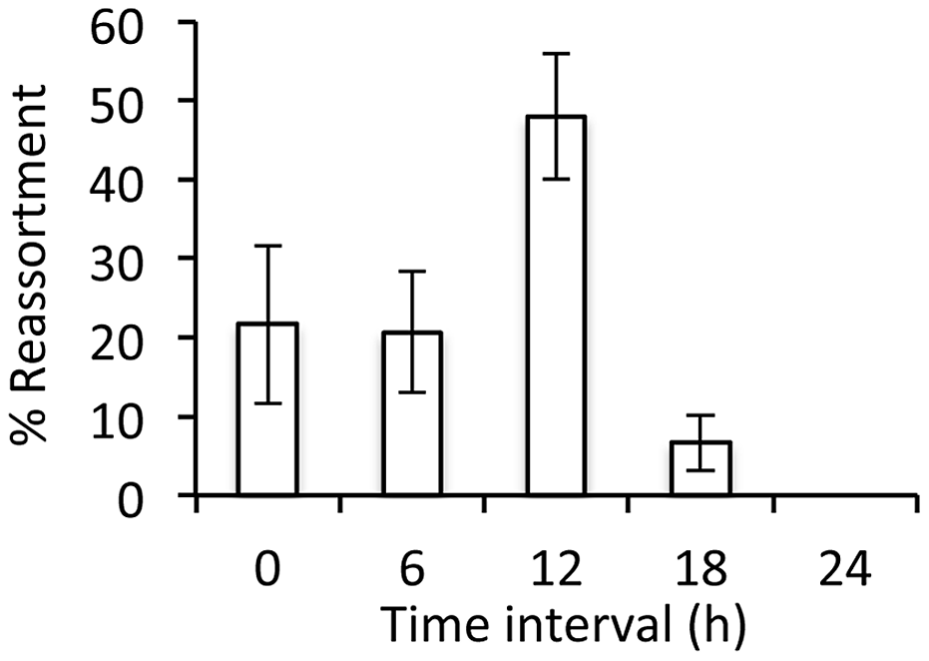
Infections separated by less than 18 h led to robust reassortment in vivo. Groups of three guinea pigs were infected with 1000 PFU rPan/99var virus and, either at the same time (0 h group), or after the indicated time interval, infected with 1000 PFU rPan/99wt virus. Plaque isolates derived from nasal washings collected 48 h after wt virus infection were genotyped by HRM analysis. The average +/− standard deviation of the percentage of isolates with reassortant genotypes is shown.

To determine how the introduction of a time interval between two influenza virus infections would impact reassortment in an animal host, we set up a similar series of infections in the guinea pig model. Groups of three guinea pigs were infected intranasally either with rPan/99var and rPan/99wt viruses together (0 h group) or with rPan/99var virus alone, followed by rPan/99wt virus 6, 12, 18 or 24 h later. Nasal washings were collected 48 h after the rPan/99wt virus infection. Since the induction of an antiviral response following primary infection has the potential to block secondary infection, we first evaluated whether the rPan/99wt virus infection was productive by quantifying wt and var HA vRNA in each nasal wash sample. In all groups, rPan/99wt virus was found to infect productively (Table 2); in the 24 h group, however, Ct values for HAwt were found to be markedly higher than in the other groups, indicating a less robust rPan/99wt virus infection in these animals. In a separate experiment, infection with rPan/99wt virus 48 or 72 h after rPan/99var virus did not result in productive infection (HAwt Ct>35 in 5/6 guinea pigs). Progeny virus isolates obtained from the nasal wash samples were then genotyped and scored as wt, var or reassortant. The results indicate that a delay of up to 12 hours between primary and secondary infections with rPan/99 virus does not reduce reassortment frequency (Table 2). In fact, higher levels of reassortment were seen in guinea pigs with a 12 h interval between infections than in those infected with both viruses simultaneously (p = 0.02, Student's t-test; Figure 6). In contrast, secondary infection 18 h after primary infection resulted in a low frequency of reassortant progeny (6.7% on average), whereas no reassortants were detected when the two infections were staggered by 24 h. Thus, while a brief delay between primary and secondary influenza virus infections may actually increase the potential for reassortment, no reassortment was seen with a delay of 24 h or more.

**Table 2:** Super-infection up to 12 h after primary infection leads to robust reassortment in vivo.

Guinea pig no.	Time interval (h)	Prevalence of HA RNA in bulk nasal wash fluid (Ct value)^1^		Genotypes of virus isolates (%)^2^		
		HAwt	HAvar	Reassortant	wt	var
1	0^3^	25.0	24.6	14	52	33
2	0	26.0	25.6	18	27	55
3	0	25.3	26.4	33	57	10
4	6	28.1	27.1	29	29	43
5	6	26.2	26.8	14	64	23
6	6	24.8	25.2	19	62	19
7	12	25.4	25.6	42	33	26
8	12	27.2	26.7	45	23	32
9	12	26.2	24.6	57	0	43
10	18	29.1	23.3	10	5	86
11	18	25.7	23.0	5	24	71
12	18	29.8	23.2	5	0	95
13	24	31.5	22.7	0	0	100
14	24	33.7	26.0	0	0	100
15	24	32.5	25.8	0	0	100
4wt	n/a	27.9	>40	n/a	n/a	n/a
4var6	n/a	>40	25.9	n/a	n/a	n/a

1 Quantitative PCR was performed using primers specific for wild-type or var virus HA segments. Average of two replicates is shown. Ct values <35 were considered indicative of productive infection.

2 n = 19–22 virus isolates from each guinea pig.

3 The proportion of wt and var viruses present in the inoculum for the 0 h group was 61% and 39%, respectively (n = 25).

4 wt and var6 controls are nasal washes collected from guinea pigs singly infected with each virus.

### Viral spread over time leads to increased numbers of co-infected cells

**Figure 7:**
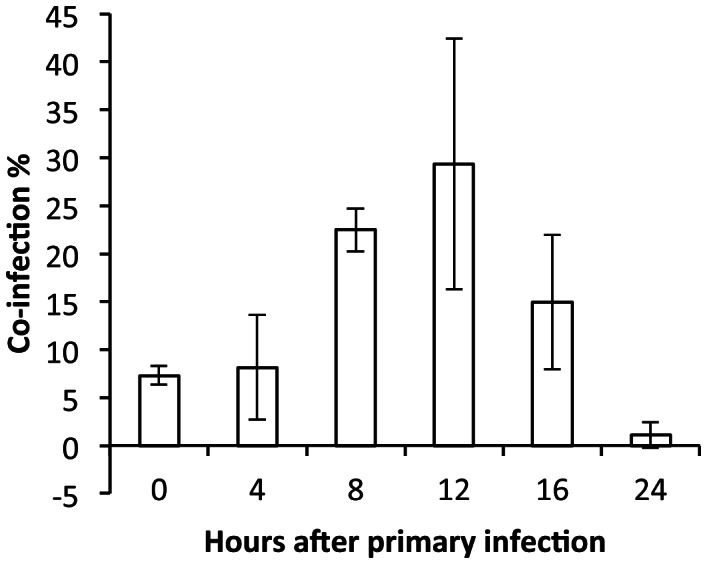
Viral spread in cell culture leads to increased frequency of co-infected cells when super-infection is delayed by 8 or 12 hours. MDCK cells were infected in the presence of trypsin at an MOI of 0.01 PFU/cell with each virus, simultaneously (0 h), or first with the var virus and then with the wt virus at the times indicated. Cell monolayers were collected and processed for flow cytometry 12 h after the wt virus infection. The average (n = 3) percentage of infected cells that were co-infected is plotted. Error bars indicate standard deviation.

We had expected co-infection rates in cell culture and in vivo to decline with increasing time interval between infections. While this was found to be the case at high MOI in MDCK cells, co-infection was increased in guinea pigs with the introduction of a 12 h delay prior to super-infection. To explain this observation, we hypothesized that the delay allows the first virus to undergo one round of replication and then spread, thereby increasing the probability that the second infection will result in doubly infected cells. We tested this hypothesis by performing an MDCK cell based co-infection experiment from a low MOI (0.01 PFU/cell) in the presence of trypsin. Under these conditions that allow for viral spread, the proportion of infected cells that were co-infected increased with an 8 or 12 h interval between inoculations (p<0.0001, chi-squared test). A decline in co-infected cells was then seen with 16 and 24 h intervals, indicating that super-infection interference has taken effect at these times after the primary infection (Figure 7).

## Discussion

We have evaluated, to our knowledge for the first time, the efficiency with which influenza A viruses undergo reassortment in the absence of fitness differences among parental and progeny genotypes. Our results show that reassortment is very efficient: where high rates of co-infection are achieved, high frequencies of reassortant genotypes are seen. These findings demonstrate that compartmentalization within the cell does not prevent extensive mixing of gene segments from two co-infecting viruses. It remains unclear whether this mixing occurs in the cytoplasm upon virus uncoating, in the nucleus during replication, during nuclear export and trafficking to the cell membrane, during the assembly process or throughout the virus life cycle. It is clear, however, that at least one stage of the life cycle allows for unrestricted exchange of gene segments.

The efficiency of reassortment was decreased when inoculation with var and wt viruses was staggered by 12 h in cell culture: despite the fact that an appreciable number of cells were co-infected under these conditions, reassortment levels were near the limit of detection. This observation suggests that reassortment is limited when the life cycles of co-infecting viruses are at markedly asynchronous. Given that released virus was sampled 12 h after the second inoculation, however, there was enough time for the second virus to undergo a full cycle of replication [31], sampling all stages of the life cycle. Thus, perhaps the predominance of progeny carrying full var genotypes was simply due to a predominance of var genomes within the co-infected cells. Higher intracellular levels of var gene segments compared to wt would be expected, given that their replication began several hours before the first wt genomes entered the cell.

A system for the study of influenza virus co-infection rates in cell culture was recently described by Bodewes et al. [32]. Two similar recombinant influenza viruses that encoded different fluorescent proteins in the place of neuraminidase (NA) were generated. Using these viruses, co-infection of MDCK cells at an MOI of 3 PFU/cell resulted in double infections in about 10% of cells. In contrast, at an MOI of approximately 1 PFU/cell, we observed that about 65% of MDCK cells were co-infected with rPan/99wt and rPan/99var viruses. Variation in experimental design most likely accounts for these differences: the efficiency of infection with the fluorescein encoding viruses may have been decreased by the absence of NA on the virions [33] and also may have differed from that seen with the rPan/99 viruses due to the H5 subtype background that was used. Deviations of our own co-infection rates from those predicted by the Poisson distribution most likely result from antiviral factors present in undiluted virus stocks used for MOI = 10 infections, the contribution of gene segments from non-infectious particles (especially important at lower MOIs), and routine experimental error.

The proportion of progeny viruses with reassortant genotypes was found to increase exponentially with the proportion of cells that were co-infected. This relationship may reflect the relative likelihood at each MOI of an individual cell receiving equal doses of wt and var. At low MOI, those cells that are co-infected are highly likely to have only one copy of each genome and therefore a 1∶1 ratio of wt and var. At intermediate MOI, in contrast, the probability of two wt and one var virus (or vice versa) entering a given cell is appreciable. At high MOI, virtually all cells are expected to have several copies of each genome so that the wt∶var ratios are likely to be close to 1∶1 (e.g. under MOI = 10 conditions, 9 wt and 11 var viruses might enter the same cell). Thus, the data suggest that discordant doses at the level the individual co-infected cell decrease the efficiency of reassortment.

The rates of reassortment observed in co-infected guinea pigs were markedly higher than those seen previously in a ferret model [19], but comparable to those obtained in a swine based experiment [26]. In a risk assessment study in ferrets, Jackson et al. examined the progeny arising from simultaneous co-infection with 10^5.7^ PFU each of an H3N2 seasonal strain (A/Wyoming/03/03) and a highly pathogenic avian H5N1 influenza virus (A/Thailand/16/06). On average, 8.7% of the isolates (n = 360) obtained from ferret nasal washings were reassortants [19]. Similarly, Ma et al. evaluated reassortment following simultaneous co-infection of pigs with 10^6^ PFU each of classical H1N1 and triple-reassortant H3N2 swine influenza viruses. In this case, 84.5% of virus isolates (n = 71) carried mixed genomes [26]. The relatively low frequency of reassortant progeny resulting from A/Wyoming/03/03+A/Thailand/16/06 virus co-infection might be accounted for by i) functional incompatibilites between the gene products ii) mismatch among packaging sequences [17], and/or iii) low co-infection rates resulting from differing receptor binding specificities [34]. Each of these aspects of functional mismatch may have been diminished in the swine experiment since the viruses used were both adapted to this host and the NS, M and NP genes were of classical swine origin in each case [35].

In guinea pigs infected with 10^6^ PFU of each virus, reassortment rates were similar to those obtained in cell cultures infected at individual MOIs of approximately 1 PFU/cell. The two situations should not be compared directly, however, since the MDCK based experiments were limited to a single cycle of replication (both by the absence of trypsin and the early time point at which samples were collected), while multiple rounds of replication occurred in vivo. Amplification of both the input viruses (leading to more opportunity for co-infection) as well as the reassortant progeny viruses may have contributed to the high frequency of reassortant genotypes. It should be noted, however, that the reassortant genotypes identified in vivo were, in general, diverse. In other words, the reassortant isolates identified were not members of one or a few clonal populations.

Influenza virus infection triggers a number of effects that disfavor super-infection. These include the induction of cellular and host antiviral responses [36], the stripping of sialic acid receptors from the cell surface by viral neuraminidase [37], and the destruction of potential target cells through lysis. Since the potency or extent of each of these effects increases over time, we predicted that the opportunity for co-infection and reassortment would decrease over time after a primary infection. In cell culture, under high multiplicity conditions, this hypothesis was found to be correct. In guinea pigs inoculated with 1000 PFU, however, infections staggered by 12 h led to a higher level of reassortment than did simultaneous co-infection. A 12 h delay between first and second infections in vivo may allow for the first virus to undergo one round of replication and then spread, thereby increasing the probability that the second infection will result in doubly infected cells. This hypothesis is supported by our observation that, following low MOI infection in cell culture, time intervals of 8 and 12 h between infections increased the number of co-infected cells compared to simultaneous infection. That fewer co-infected cells and reassortant viruses were produced from infections staggered by 16 h or 18 h in MDCK cells and guinea pigs, respectively, suggests that at these times after primary infection one or more of the mechanisms of super-infection exclusion has begun to take effect.

Recent progress in the field on the mechanisms of influenza virus genome packaging have led to the hypothesis that sequence-specific interactions between RNA segments drive their selective incorporation during virion assembly [17], [38], [39], [40], [41], [42], [43], [44], [45]. It follows that, if packaging signals vary between strains of influenza A virus, a requirement for such RNA-RNA interactions would limit reassortment between divergent viruses. The regions of each segment thought to be important for packaging [41], [43], [46] were avoided in the mutagenesis of rPan/99var virus; thus, rPan/99wt and rPan/99var viruses most likely carry identical packaging signals. For this reason, our data are not expected to, and do not, reveal linkages between the segments at the RNA level. Our results do offer some insight into packaging specificity, however, in that HRM analyses of clonal isolates allowed the typing of each segment as wt or var. We did not see clear examples of isolates that carried both a wt and a var copy of a given segment. This result is in agreement with recent publications by Chou et al. and Inagaki et al., which showed respectively that eight distinct segments are packaged into one virion [40] and that homologous gene segments compete for incorporation [47].

For the experiments described, we chose to use a seasonal influenza virus representative of the human H3N2 lineage. We would, however, expect influenza viruses of other strains and subtypes to behave similarly to the Pan/99 virus. The reason is that we are essentially studying the reassortment of a virus with itself; any strain should reassort well with itself under the “baseline” conditions described. When conditions are altered from the baseline, however, certain strain specific effects would be expected to arise. Two such effects relevant to the results herein are i) the role of receptor binding specificity and other host-adaptive traits in determining the efficiency of infection and therefore co-infection in a given host species or target cell type; and ii) the precise timing with which super-infection interference takes effect. The latter will most likely vary with the rate of viral growth and may hinge on the efficiency of IFN suppression or stripping of cellular sialic acids, depending on the mechanism(s) at play.

Much remains to be done to gain a comprehensive understanding of influenza virus reassortment and the conditions under which it occurs. In addition to the effects of dose and timing described herein, cell tropism, host species, pre-existing immunity in the host, relative rates of viral growth and a wide range of strain specific factors that contribute to the phenomenon of segment mismatch will each come into play in determining the outcome of mixed influenza virus infections. In turn, the number of virus particles transmitted from a co-infected host to a recipient will be important in determining the epidemiological significance of reassortment events. Comparison of intra- and inter-host genetic diversity in equine and swine influenza suggests that the transmission bottleneck is loose; in other words, the diversity of genotypes transmitted was similar to that present in the initial host [48], [49], [50]. Under these conditions, a reassortant virus present even as a minor population would frequently be passed on to additional hosts.

In sum, our findings indicate that influenza virus reassortment is an efficient process in a co-infected cell and in a co-infected host, and that the frequency of reassortment both at the level of individual cells as well as that of the animal host is dependent on dose and timing of infection. In establishing a simplified and well-controlled system to examine reassortment, we have laid the groundwork for future studies that will focus on virus and host-specific factors. By systematically varying individual parameters within our system, we hope to quantify the impact of a wide range of factors such that the complexity of influences determining natural reassortment rates can be evaluated for a given situation.

## Materials and Methods

### Ethics statement

This study was performed in accordance with the recommendations in the Guide for the Care and Use of Laboratory Animals of the National Institutes of Health. Animal husbandry and experimental procedures were approved by the Emory University Institutional Animal Care and Use Committee (IACUC protocol #2000719).

### Cells

Madin-Darby Canine Kidney (MDCK) cells were maintained in minimum essential medium (MEM) supplemented with 10% FBS and penicillin-streptomycin. 293T cells were maintained in Dulbecco's MEM supplemented with 10% FBS.

### Guinea pigs

Female, Hartley strain, guinea pigs weighing 300–350 g were obtained from Charles River Laboratories. Prior to intranasal inoculation, nasal lavage or CO_2_ euthanasia, guinea pigs were sedated with a mixture of ketamine and xylazine (30 mg/kg and 2 mg/kg, respectively). Inoculation and nasal lavage were performed as described previously [51], with PBS as the diluent/collection fluid in each case.

### Viruses

rPan/99wt and variant viruses were recovered by reverse genetics following standard procedures [52], [53]. Briefly, a 12 plasmid rescue system based on pPOL1 and pCAGGS vectors and co-culture of 293T and MDCK cells were used. Plaque isolates derived from rescue supernatants were amplified in 11-day-old embryonated chicken eggs to generate virus stocks and stock titers were determined by plaque assay on MDCK cells.

Four rPan/99 based viruses were used in the research described: rPan/99wt, rPan/99var6, rPan/99wt-HIS and rPan/99var2-HA. The first is a reverse genetics derived version of the wild-type A/Panama/2007/99 virus. The second, rPan/99var6, contains the following silent mutations relative to rPan/99wt virus (nucleotide numbering is from the 5′ end of the cRNA): NS C329T, C335T, and A341G; M C413T, C415G and A418C; NA C418G, T421A and A424C; NP C537T, T538A and C539G; HA T308C, C311A, C314T, A464T, C467G and T470A; PA A342G and G333A; PB1 C288T and T297C; and PB2 C354T and C360T. The third, rPan/99wt-HIS, differs from rPan/99wt virus only in that it encodes a HIS tag within the HA open reading frame (inserted immediately after the sequence encoding the signal peptide [54]). The fourth, rPan/99var2-HA, encodes an HA tag within the HA open reading frame (again, inserted after the signal peptide) as well as the following silent mutations: NS C329T, C335T, and A341G; M C413T, C415G and A418C; NA C418G, T421A and A424C; NP C537T, T538A and C539G; HA T308C, C311A, C314T, A464T, C467G and T470A; PA G603A, T604A and C605G; PB1 C346T, T348G and A351G; and PB2 T621C, T622A and C623G. The rPan/99wt-HIS and rPan/99var2-HA viruses were used in the MDCK cell base experiments described, whereas the rPan/99wt and rPan/99var6 viruses were used in the guinea pig experiments.

Our rationale for the above-described mutagenesis is as follows. The mutations were selected to allow differentiation of wt and var viruses by HRM analysis of ∼100 bp amplicons containing the mutation sites. In addition, we wished to avoid attenuation of the var viruses and therefore used a minimal number of mutations and avoided any known cis-acting signals. Two or three mutations were found to be sufficient to obtain clear HRM results, and C/T and A/G mutations were preferred since they confer the greatest change in melting properties. In addition, a second set of three mutations was introduced into the var HA segment such that the two mutated regions could be used as primer binding sites unique to wt or var so that these segments could be quantified in a standard qPCR assay (as reported in Table 2).

### Determination of reassortment frequency in vitro following simultaneous co-infection

rPan/99wt-HIS and rPan/99var2-HA viruses were mixed in equal proportions and then diluted with PBS to the appropriate titer for inoculation at MOI 10, 1, 0.1 or 0.01 PFU/cell of each virus. For the calculation of MOI, each well of a 6-well dish was assumed to have 1×10^6^ cells. Prior to inoculation of MDCK cells, growth medium was removed, monolayers were washed with PBS three times and the 6-well dish was placed on ice. Each well was inoculated with a 250 ul volume and cells were incubated at 4°C for one hour to allow equal binding of all virus. Unattached virus was removed by aspirating inoculum and washing three times with PBS. After the addition of virus medium (MEM supplemented with 3% BSA, Penicillin Streptomycin, and 1 ug/ml trypsin where indicated), cells were transferred to 33°C. At 12 hours post-infection, supernatant was collected and stored at −80°C for subsequent genotyping of released virus. MDCK-infected cells were harvested and prepared for flow cytometry (see below). To determine the gene constellations of viruses present in the supernatant, 121 (Figure 3) or 21 (Figure 4) clones per sample were isolated by plaque assay of the supernatant and all 8 vRNA segments from each was typed by HRM analysis.

### Determination of reassortment frequency in vitro following super-infection

Seven 6-well plates of MDCK cells were infected in triplicate for this experiment. For the first plate (“0 h” time point), MDCK cells were infected with rPan/99wt-HIS and rPan/99var2-HA viruses as described above for the simultaneous co-infection at MOI 10 of each virus. The remaining plates were each infected at MOI 10 of rPan/99var2-HA virus alone at time = 0 h and then infected subsequently (2, 4, 8, 12, 16, or 24 h after the var virus infection) with rPan/99wt-His virus at MOI 10. In each case, infections were carried out on ice and with a 1 h attachment period at 4°C, as described above. Supernatant was collected from each plate at 12 h following infection with rPan/99wt-His virus and stored at −80°C. Cells were harvested at this same time point and prepared for flow cytometry (see below). The frequency of reassortant progeny in the supernatant was determined by typing all 8 gene segments of 21 plaque isolates from each sample by HRM analysis.

### Enumeration of infected and co-infected cells

To determine the number of infected and co-infected cells, MDCK cells were harvested 12 h after either wt/var virus co-infection or 12 h after wt virus infection by trypsinizing the monolayer and collecting with serum-supplemented medium. The cells were then washed 3 times with PBS-2% fetal calf serum (FBS) and incubated with Penta HIS Alexa Fluor 647 conjugated antibody (5 ug/ml; Qiagen) and Anti-HA-FITC Clone HA-7 (7 ug/ml; Sigma Aldrich) for 45 minutes, on ice. Cells were then washed 2 times with PBS-2% FBS and re-suspended with 200 ul of PBS-2% FBS and 5 ul/sample (0.25 ug) of 7-Amino Actinomycin D (7-AAD), a dead cell excluder (BD Biosciences). Flow cytometry was performed using a FACSVerse flow cytometer (Becton Dickinson) and analyzed with FlowJo software.

### Determination of reassortment frequency in vivo following simultaneous co-infection

rPan/99wt and rPan/99var6 viruses were mixed in equal proportions and then diluted in PBS to the appropriate titer (6.6×10^3^ or 6.6×10^6^ total PFU/ml for inoculation with 10^3^ or 10^6^ PFU of each virus, respectively). A total of five guinea pigs per dose were inoculated intranasally with 300 ul and unused inoculum was stored at −80°C. At 48 h post-infection nasal washings were collected and stored at −80°C. To determine the ratio of wt and var viruses present in the inoculum, 25–30 clones were isolated by plaque assay of the unused inoculum and two vRNA segments from each was typed by HRM analysis. Similarly, the frequency of reassortant progeny shed into nasal washings was determined by typing all eight gene segments of 19–24 plaque isolates derived from each nasal wash sample by HRM analysis. The data shown in Table 1 are the products of three different experiments: animals 1, 2, 3, 6, 7, and 8 comprised one experiment; animals 4 and 5 comprised a second; and animals 9 and 10 comprised a third.

### Determination of reassortment frequency in vivo following super-infection

Five groups of three guinea pigs were used in this experiment. The first group (the “0 h” group) was infected with rPan/99wt and rPan/99var6 viruses as described above for the simultaneous co-infection with 10^3^ PFU of each virus. The remaining guinea pigs were each infected with 10^3^ PFU of rPan/99var6 virus alone at time = 0 h and then infected subsequently (6, 12, 18 or 24 h after the var virus infection) with 10^3^ PFU of rPan/99wt virus. Nasal washings were collected from each guinea pig 48 h following infection with the rPan/99wt strain and stored at −80°C. The frequency of reassortant progeny shed into nasal washings was determined by typing all eight gene segments of 19–22 plaque isolates derived from each nasal wash sample by HRM analysis.

### Determination of virus genotypes by high resolution melt analysis

To screen virus released from co-infected cell cultures or guinea pigs, the following steps were performed. 1) Plaque isolates were obtained by plaque assay of cell culture supernatants or guinea pig nasal wash fluids in 10 cm cell culture dishes. Agar plugs above well separated (>0.5 cm apart) plaques were picked using 5 ml serological pipettes, ejected into 160 ul of PBS in 1.5 ml tubes, and then stored at −80°C. 2) RNA was extracted from the agar plugs using the Qiagen QiaAmp Viral RNA kit, with the following modifications to the manufacturer's protocol: carrier RNA was not used, agar plugs in PBS were heated at 65°C for 5 min prior to mixing with AVL lysis buffer, and 40 ul water was used for the elution step. 3) Twelve microliters of RNA was reverse transcribed using Maxima reverse transcriptase (Fermentas) according to the manufacturer's instructions. 4) cDNA was used as template in qPCR reactions. Four microliters of 1∶4 diluted cDNA were combined with the appropriate primers (0.4 uM final concentration; see Supplementary Table S1 for primer sequences) and Precision Melt Supermix (BioRad) in wells of a white, thin wall, 384 well plate (BioRad). qPCR and melt analyses were carried out in a CFX384 Real-Time PCR Detection System, as per the instructions provided with the Precision Melt Supermix. Data were analysed using Precision Melt Analysis software (BioRad). Viruses were scored as reassortant if the genome comprised a mixture of wt and var gene segments in any proportion (e.g. both 7∶1 reassortants and 4∶4 reassortants were treated in the same way). Occasionally, one gene segment from a given isolate could not be typed as wt or var with high confidence (this was the case with approximately 2.5% of segments); such isolates were scored as wt or var parental viruses if all other gene segments were wt or var, respectively. If greater than one segment could not be typed, the isolate was excluded from the analysis.

### Quantification of HA segment in bulk nasal wash fluids

The HA segments of rPan/99 wt and rPan/99var6 viruses differ by 6 nucleotides in two clusters: T308C/C311A/C314T and A464T/C467G/T470A. Forward and reverse primers encompassing these mutation clusters were designed: HAwt 295F/HAwt 481R and HAvar 295F/HAvar 481R. These primers specifically amplify the wt or var HA segments, respectively, allowing their quantification by conventional qPCR methods. Thus, RNA extracted directly from nasal lavage fluids was subjected to reverse transcription followed by qPCR using SsoFast Evagreen Supermix (BioRad), according to the manufacturer's instructions. qPCR was performed with a CFX384 Real-Time PCR Detection System and results were analysed using CFX Manager software (BioRad).

### Statistical analyses

A one-sided exact test was applied to the data shown in Figure 3 to determine whether the proportion of isolates with parental genotypes was statistically greater than 2/256, or 0.008 (the expected value if reassortment occurred with full efficiency). Two-sided Fisher's exact tests were used to compare proportions of reassortant vs. parental progeny for data shown in Figures 4 and 5, while chi-squared tests were applied to compare proportions of singly vs. doubly infected cells for flow cytometric data shown in Figures 4, 5 and 7. Finally, unpaired, two-sided Student's t-tests were applied to data shown in Figure 6 and Tables 1 and 2.

## Supporting Information

Table S1 **Nucleotide sequences of primers used for HRM analysis.**
(DOCX)
