# Supplementary material for: Influenza Virus Reassortment Occurs with High Frequency in the Absence of Segment Mismatch
Source: PLoS Pathog. 2013 Jun 13;9(6):e1003421. doi: 10.1371/journal.ppat.1003421 (PMC3681746; doi:10.1371/journal.ppat.1003421)
Supplement: Table S1 — Nucleotide sequences of primers used for HRM analysis. (DOCX) [file ppat.1003421.s001.docx]

Supplementary Table 1. Nucleotide sequences of primers used for HRM analysis.

| Virus^1^ | Segment | Silent mutations | Forward Primer | Reverse Primer |
| --- | --- | --- | --- | --- |
| rPan/99var | NS | C329T, C335T, A341G | acctgcttcgcgatacataac | aggggtccttccactttttg |
| rPan/99var | M | C413T, C415G, A418C | gcactcagttattctgctggtg | aatgccacttcggtggttac |
| rPan/99var | NA | C418G, T421A, A424C | tcatgcgatcctgacaagtg | tgtcatttgaatgcctgttg |
| rPan/99var | NP | C537T, T538A, C539G | caacataccagaggacaagagc | accttctagggagggtcgag |
| rPan/99var | HA | T308C, C311A, C314T, A464T, C467G, T470A | ccttgatggagaaaactgcac | caacaaaaaggtcccattcc |
| rPan/99var-2-HA | PA | G603A, T604A, C605G | caagaaatggccaacagagg | tgcgcatagttcctgagattt |
| rPan/99var-2-HA | PB1 | C346T, T348G, A351G | gctatggccttccttgaaga | tgtccacccttgtttgttga |
| rPan/99var-2-HA | PB2 | T621C, T622A, C623G | aaagaagaactccgagattgc | ttgttccgccagcaactg |
| rPan/99var-6 | PA | A342G, G333A | tgcaacactactggagctgag | ctccttgtcactccaatttcg |
| rPan/99var-6 | PB1 | C288T, T297C | aacccaattgatggaccact | gatccctgggtgggattc |
| rPan/99var-6 | PB2 | C354T, C360T | tggaatagaaatggacctgtga | ggttccatgttttaacctttcg |

^1^ Primers were used to differentiate the segments of the indicated var virus from those of the wt virus. Since the NS, M, NA, NP and HA segments of rPan/99var2-HA and rPan/99var6 viruses are identical, the generalized term rPan/99var is used.
